# Effects of 2018 Japan floods on healthcare costs and service utilization in Japan: a retrospective cohort study

**DOI:** 10.1186/s12889-023-15205-w

**Published:** 2023-02-08

**Authors:** Shuhei Yoshida, Saori Kashima, Yuji Okazaki, Masatoshi Matsumoto

**Affiliations:** 1 Department of Community-Based Medical System, Graduate School of Biomedical and Health Sciences, Hiroshima University, 1-2-3 Kasumi, Minami-ku, 734-8551 Hiroshima-shi, Hiroshima-ken, Japan; 2 Department of General Internal Medicine, Hiroshima University Hospital, 1-2-3 Kasumi, Minami-ku, 734-8551 Hiroshima-shi, Hiroshima-ken, Japan; 3 Planetary Health and Innovation Science Center, IDEC Institute, Hiroshima University, 1-3-2 Kagamiyama, Higashi-Hiroshima-shi, Hiroshima-ken, Japan; 4 Environmental Health Sciences Laboratory, Graduate School of Advanced Science and Engineering, Hiroshima University, 1-3-2 Kagamiyama, Higashi-Hiroshima-shi, Hiroshima-ken, Japan

**Keywords:** Climate change, Natural disaster, Healthcare cost, Healthcare service utilization

## Abstract

**Background:**

Floods and torrential rains are natural disasters caused by climate change. Unfortunately, such events are more frequent and are increasingly severe in recent times. The 2018 Japan Floods in western Japan were one of the largest such disasters. This study aimed to evaluate the effect of the 2018 Japan Floods on healthcare costs and service utilization.

**Methods:**

This retrospective cohort study included all patients whose receipts accrued between July 2017 and June 2019 in Hiroshima, Okayama, and Ehime prefectures using the National Database of Health Insurance Claims. We used Generalized Estimating Equations (GEEs) to investigate yearly healthcare costs during the pre-and post-disaster periods, quarterly high-cost patients (top 10%), and service utilization (outpatient care, inpatient care, and dispensing pharmacy) during the post-disaster period. After the GEEs, we estimated the average marginal effects as the attributable disaster effect.

**Results:**

The total number of participants was 5,534,276. Victims accounted for 0.65% of the total number of participants (n = 36,032). Although there was no significant difference in pre-disaster healthcare costs (p = 0.63), post-disaster costs were $3,382 (95% CI: 3,254–3,510) for victims and $3,027 (95% CI: 3,015–3,038) for non-victims (p < 0.001). The highest risk difference among high-cost patients was 0.8% (95% CI: 0.6–1.1) in the fourth quarter. In contrast, the highest risk difference of service utilization was in the first quarter (outpatient care: 7.0% (95% CI: 6.7–7.4), inpatient care: 1.3% (95% CI: 1.1–1.5), and dispensing pharmacy: 5.9% (95% CI: 5.5–6.4)).

**Conclusion:**

Victims of the 2018 Japan Floods had higher medical costs and used more healthcare services than non-victims. In addition, the risk of higher medical costs was highest at the end of the observation period. It is necessary to estimate the increase in healthcare costs according to the disaster scale and plan for appropriate post-disaster healthcare service delivery.

**Supplementary Information:**

The online version contains supplementary material available at 10.1186/s12889-023-15205-w.

## Background

Natural disasters caused by climate change are a significant problem worldwide [1]. These disasters include heatwaves, storms, and floods, which have destroyed safe living environments and caused death and various diseases [2, 3]. To recover from the damage caused by disasters, affected people and regions consume enormous healthcare resources and costs due to the need for medical care immediately after a disaster and over a subsequent long period of recovery [4]. Therefore, the World Health Organization (WHO) declared that studies on the cost estimation of such disasters should be prioritized and are crucial for developing plans for disaster preparedness policies [5].

Japan has also been affected by climate-related disasters, especially floods and torrential rains. The most severe recent event was the 2018 Japan Floods (2018-*nen-sitigatu-gou*) in western Japan in July 2018 [6–8]. The impact included 237 fatalities, 8 missing persons, 433 injured persons, and 6,767 houses completely destroyed [9, 10]. Although the main affected areas were in western Japan, heavy rains have been recorded in various regions throughout Japan [11]. This disaster caused wide damage compared with other designated disasters of extreme severity from 2017 to 2022 [12]. In addition, the damage caused by the 2018 Japan Floods was further extended due to other disasters, such as earthquakes, which subsequently occurred in the same areas [11]. The total damage cost of this disaster was approximately US$9.86 billion (\1,158 billion), which was 2.6% of the Japanese nominal GDP in 2018 [13, 14]. This was the highest damage cost of all torrential rains or floods in Japan’s history. However, this amount does not include healthcare costs. There are two major insurance schemes in Japan to provide healthcare [15, 16]. These include health and long-term care insurance. We have already examined the change in long-term care services and costs [17]. This study showed that an increase in LTCI costs and utilization of facilities where older people could stay in could decrease the utilization of services that they avail when staying in private homes. To clarify the total health-related costs of this disaster, it is necessary to identify excess expenditures for healthcare services provided by health insurance during and after a disaster.

The direct health impacts of torrential rain and floods include death or injury due to drowning, electric shock, and other accidental causes [18, 19]. Moreover, the loss of clean water supply, interruption of transportation networks, shutdown of electric power, and impairment of communication networks cause indirect health impacts, such as the exacerbation of mental illness and cognitive impairment [17, 20–23]. Previous studies that examined the increase in healthcare service utilization and healthcare costs after disasters have been either ecological studies or investigations of a few specific affected municipalities or facilities [24, 25]. After the 2018 Japan Floods, local governments individually certified victims according to the damage sustained [26]. As such, it is possible to identify victims affected by this disaster at an individual level and assess their healthcare impacts in more detail.

Japan has universal health coverage that covers almost all the necessary medical care [16]. Therefore, it is possible to totally evaluate healthcare service utilization and costs, including outpatient care, inpatient care, and dispensing pharmacies. Accordingly, this study examined the change in healthcare service utilization and costs among victims of the 2018 Japan Floods. We investigated the impact on the healthcare system and discussed preparedness for future climate change-related disasters.

## Methods

### Study design

This study was a retrospective cohort study.

### Universal health insurance coverage

In 1961, Japan introduced universal health insurance coverage [16]. The copayment rate of the most insured persons was 30%. People aged 75 years and older with incomes below those of average workers pay 10% as copayment. People aged 70 to 74 years, with incomes below the average, and children younger than 6 years old pay 20% as the copayment. Many prefectures and municipalities have established their own medical expense subsidy programs to reduce some or all of the copayment for children. When the monthly copayment is higher than a certain amount set by the insured person, high-cost medical expense benefits subsidize the portion that exceeds that amount.

### Data source

This study was conducted using the National Database of Health Insurance Claims (NDB), an administrative claims database in Japan. This database contains age classification, sex, and healthcare facilities used by patients. Diagnosed disease name, socioeconomic status, and address were not included. The receipt data are stored in the NDB every month, 3 months after the use of healthcare facilities [27]. Although the NDB contains almost all the information on medical services in Japan, those who receive public income support or those covered through automobile liability insurance or workers’ compensation insurance are not included in the NDB. The exclusion proportion was < 2%. We obtained permission to use a part of the NDB data as special sampling from the Ministry of Health, Labour and Welfare (approval no. 1223-2). Using this dataset, it was found that the victims used more of several types of medicines than non-victims [14–17].

### Setting

The settings were Hiroshima, Okayama, and Ehime prefectures. These prefectures belong to the Setouchi climatic zone and are relatively warm and dry areas with an average temperature of approximately 15 °C and an annual average rainfall of approximately 1,000–1,600 mm [28]. In the past, there were no major floods or torrential rains; however, in 2014, localized torrential rains caused landslides in Hiroshima Prefecture. Although this disaster was far smaller than the 2018 Japan Floods, it was reported to occur with a probability of once every 100 years [29]. Four years after this disaster, the 2018 Japan Floods hit mainly western Japan. Hiroshima, Okayama, and Ehime prefectures incurred a large proportion of the damage caused by the disaster: 212 out of 237 deaths, 8 out of 8 missing cases, 6,603 out of 6,767 houses completely destroyed, and 10,012 out of 11,243 houses partially destroyed [10].

### Participants

We detected 5,625,341 individuals in the database. All patients whose medical receipts accrued once between July 2017 and June 2019 were included as participants. Because this disaster brought torrential rains from June 28, 2018, to July 8, 2018, the period was from one year before the disaster to one year after the disaster.

### Variables

#### Objective variables

The objective variables were total medical costs and healthcare service utilization: outpatient care, inpatient care, and dispensing pharmacies. Total medical costs were calculated from the total units of medical, Diagnosis Procedure Combination, and dispensing pharmacy receipts of the participants, and summed for one year before and after the disaster. We used the average currency exchange rate for 2018 (1 US dollar = 110 Japanese yen) to convert Japanese yen to US dollars [30]. In addition, to examine changes after the disaster, all medical costs were calculated per quarter (Quarter 1: July 2018 to September 2018, Quarter 2: October 2018 to December 2018, Quarter 3: January 2019 to March 2019, Quarter 4: April 2019 to June 2019). The quarterly medical costs contained the excess 0, which causes bias when treated as a continuous variable [31]. Additionally, it has been reported that some patients intensively consume the majority of the medical costs as high-cost patients [32]. Therefore, quarterly medical costs were categorized into binary variables (top 10%, 30%, and 50%). In addition, whether a participant used a healthcare service (outpatient care, inpatient care, and dispensing pharmacy) was classified as a binary variable by each quarter.

#### Main exposure

The main exposure was disaster status. Local governments certified individuals as victims according to the assessed damage from the disaster. Based on this certification, we divided the participants into victims and non-victims as the main exposure groups. The certification criteria are as follows as with previous studies: (1) a person’s house was completely or partially damaged, burned down, flooding of a floor, or similar damage; or (2) the main breadwinner died, was seriously injured, became ill, or went missing [17, 22, 23, 33]. The disaster status tagged for each subject in the NDB was extracted for this study. Insured persons certified as victims were exempt from copayments for medical services.

#### Covariates

We extracted the sex and age classification codes. In addition, we specified the prefecture in which a participant most frequently accessed medical services (Okayama, Hiroshima, or Ehime prefecture).

### Statistical analysis

#### Descriptive analysis

Here, we describe the baseline characteristics of the victims and non-victims. The Chi-square test was used for discrete variables. We performed Wilcoxon’s rank-sum test for ordinal and continuous variables that were not normally distributed.

Yearly total healthcare costs between victims and non-victims.

To compare yearly total healthcare costs between victims and non-victims, we used Generalized Estimating Equations (GEEs). The objective variable is the yearly total healthcare costs from one year before the disaster to one year after the disaster. To examine the change in time course during the observation period, we added the interaction term between disaster status and year, along with disaster status, year, age classification, and sex as covariates. We specified the GEE models with a gamma distribution and a log-linked function. In the GEE models, we considered correlations among participants using exchangeable correlation structures.

#### Quarterly total healthcare costs and service utilization

We also used GEE models to examine the association between disaster status and quarterly total healthcare costs (the top 10%, 30%, or 50%). The period of each quarter was as follows: quarter 1 was from July 2018 to September 2018; quarter 2 was from October 2018 to December 2018; quarter 3 was from January 2019 to March 2019; and quarter 4 was from April 2019 to June 2019. The interaction term between disaster status and a quarter was adopted as a covariate, along with disaster status, quarter, age classification, and sex. We specified the GEE models using a Poisson distribution, log-linked function, and robust error variances. To analyze healthcare service utilization for outpatient care, inpatient care, and dispensing pharmacies, we also performed analyses using the same GEE models. All participants were included in the models.

We showed the chronological changes in the population-averaged effects of the disaster as Predictive Margins (PMs) using the interaction term between disaster status and quarter. We used the results of the GEEs to show these estimations. Using the results from GEEs, we estimated the disaster-attributable impact for each year or quarter as the PMs. The PMs were calculated for victims and non-victims as the sample-weighted average of the predicted responses from the GEE models, keeping all other covariates fixed.

All statistical analyses were performed using STATA/MP version 16 (StataCorp 2019).

## Results

Table 1 shows the participant characteristics. The total number of participants in the study was 5,534,276. Victims accounted for 0.65% of the total number of participants (n = 36,032). The victims were older and consisted of more women than non-victims (p < 0.001). The percentage of victims who belonged to Okayama prefecture was 47% (n = 16,921). The annual and quarterly medical costs of victims were higher than those of non-victims (p < 0.001). Regarding healthcare service utilization, outpatient care was highest in the third quarter, with 84.6% (n = 30,495) for victims and 77.8% (n = 4,279,071) for non-victims. The utilization of inpatient care was highest in the first quarter, with 6.7% (n = 2,431) for victims and 4.2% (n = 229,607) for non-victims. Regarding the utilization of dispensing pharmacies, the third quarter was the highest, with 59% (n = 21,276) of victims and 52.8% (n = 2,902,891) of non-victims.

**Table 1:** Participant characteristics

		Victims (%)		Non-victims (%)		p value
		36,032	0.65	5,498,244	99.35	
Age classification, n (%)	0–19	4,835	13.4	1,058,674	19.3	< 0.001*
	20–39	4,646	12.9	1,094,870	19.9	
	40–59	7,726	21.4	1,338,343	24.3	
	60–79	13,669	37.9	1,452,007	26.4	
	80+	5,156	14.3	554,350	10.1	
Sex, n (%)	Men	15,982	44.4	2,554,619	46.5	< 0.001*
	Women	20,050	55.6	2,943,625	53.5	
Prefecture, n (%)	Okayama	16,921	47.0	1,520,052	27.6	< 0.001*
	Hiroshima	9,766	27.1	2,263,143	41.2	
	Ehime	6,780	18.8	1,100,447	20.0	
	Missing or others‡	2,565	7.1	614,602	11.2	
Medical costs per year (USD), mean (SD)	July 2017 to June 2018 (pre-disaster)	3,296	42.6	2,758	3.4	< 0.001†
	July 2018 to June 2019 (post-disaster)	4,100	48.0	3,029	2.4	< 0.001†
Medical costs per quarter for one year after the disaster (USD), mean (SD)	First	1,052	18.0	740	1.2	< 0.001†
	Second	1,031	16.0	767	1.2	< 0.001†
	Third	958	14.0	762	1.2	< 0.001†
	Fourth	1,057	16.0	760	1.1	< 0.001†
Top 10% per quarter for one year after the disaster, no (%)	First	5,010	13.9	548,390	10.0	< 0.001*
	Second	5,177	14.4	548,237	10.0	< 0.001*
	Third	5,100	14.2	548,306	10.0	< 0.001*
	Fourth	5,710	15.9	547,671	10.0	< 0.001*
Top 30% per quarter for one year after the disaster, no (%)	First	14,733	40.9	1,645,312	29.9	< 0.001*
	Second	15,393	42.7	1,644,756	29.9	< 0.001*
	Third	15,238	42.3	1,644,880	29.9	< 0.001*
	Fourth	15,997	44.4	1,644,244	29.9	< 0.001*
Top 50% per quarter for one year after the disaster, no (%)	First	23,052	64.0	2,743,703	49.9	< 0.001*
	Second	23,313	64.7	2,743,814	49.9	< 0.001*
	Third	22,842	63.4	2,743,974	49.9	< 0.001*
	Fourth	23,560	65.4	2,743,190	49.9	< 0.001*
Participants who did not incur any medical costs per quarter for one year after the disaster, no (%)	First	5,642	15.7	1,478,597	26.9	< 0.001*
	Second	5,112	14.2	1,386,125	25.2	< 0.001*
	Third	5,000	13.9	1,166,915	21.2	< 0.001*
	Fourth	5,321	14.8	1,359,043	24.7	< 0.001*
Service utilization of outpatient care per quarter for one year after the disaster, no (%)	First	29,897	83	3,966,137	72.1	< 0.001*
	Second	30,347	84.2	4,059,739	73.8	< 0.001*
	Third	30,495	84.6	4,279,071	77.8	< 0.001*
	Fourth	30,276	84	4,089,261	74.4	< 0.001*
Service utilization of inpatient care per quarter for one year after the disaster, no (%)	First	2,431	6.7	229,607	4.2	< 0.001*
	Second	2,089	5.8	227,513	4.1	< 0.001*
	Third	1,899	5.3	222,205	4	< 0.001*
	Fourth	2,061	5.7	223,940	4.1	< 0.001*
Service utilization of dispensing pharmacies per quarter for one year after the disaster, no (%)	First	19,928	55.3	2,501,098	45.5	< 0.001*
	Second	20,671	57.4	2,663,956	48.5	< 0.001*
	Third	21,276	59	2,902,891	52.8	< 0.001*
	Fourth	20,473	56.8	2,682,600	48.8	< 0.001*

SD: standard deviation, *: chi-squared test, †: Wilcoxon rank-sum test, ‡: Excluded from the chi-squared test, First quarter: July 2018 to September 2018, Second quarter: October 2018 to December 2018, Third quarter: January 2019 to March 2019, Fourth quarter: April 2019 to June 2019

We used the average currency exchange rate for 2018 (1 US dollar = 110 Japanese yen) to convert Japanese yen to US dollars.

Supplementary Table 1 shows the cutoff costs of the top 10%, 30%, and 50% and the percentage of total medical costs consumed [see Supplementary File 1]. Cutoff costs of the top 10%, 30%, and 50% were from $1142.8 to $1192.5, $387.6 to $415.8, and $148.0 to $186.6, respectively. The top 10% of participants consumed 71.1–73%, the top 30% of participants consumed 88.8–90.8%, and the top 50% of participants consumed 96.3–97.7% of the total medical costs.

Table 2 shows the attributable costs for the 2018 Japan Floods based on the results of the GEEs [see Supplementary File 2]. There was no significant difference in the PMs of yearly healthcare costs between the two groups in the year before the disaster after adjusting for age classification and sex. The PM was $2,734 (95% CI: 2,631–2,838) for victims and $2,761 (95% CI: 2,750–2,771) for non-victims (p = 0.63). In contrast, the PM of costs for one year after the disaster was $3,382 (95% CI: 3,254–3,510) for victims and $3,027 (95% CI: 3,015–3,038) for non-victims (p < 0.001).

**Table 2:** Predictive Margins on yearly medical costs

Year	Victims			Non-victims		
	PM (USD)	95% CI		PM (USD)	95% CI	
		Lower	Upper		Lower	Upper
July 2017 to June 2018 (pre-disaster)	2,734	2,631	2,838	2,761	2,750	2,771
July 2018 to June 2019 (post-disaster)	3,382	3,254	3,510	3,027	3,015	3,038

PM: Predictive Margin, CI: Confidence Interval

Table 3 shows the PMs as the attributable risk from the 2018 Japan Floods, estimated by the result of GEEs [see Supplementary File 3]. Victims had a significantly higher risk of becoming the top 10% in all periods (p < 0.001). The maximum increase in risk was 2.4% (95% CI: 2.1–2.7) in the fourth quarter. Disasters were also significantly associated with the top 30% and 50%. According to the PMs, the risk difference between victims and non-victims was higher during all quarters after adjusting for age classification and sex (p < 0.001). Moreover, the risks of becoming the top 10%, 30%, and 50% increased gradually throughout the quarters.

**Table 3:** Predictive Margins on quarterly total medical costs (%)

	Quarter	Victims			Non-victims		
		PM (%)	95% CI		PM (%)	95% CI	
			Lower	Upper		Lower	Upper
Top 10% per quarter for one year after the disaster	First	10.8	10.6	11.1	10.0	10.0	10.0
	Second	11.2	10.9	11.5	10.0	10.0	10.0
	Third	11.0	10.8	11.3	10.0	10.0	10.0
	Fourth	12.3	12.1	12.6	10.0	10.0	10.0
Top 30% per quarter for one year after the disaster	First	33.4	33.0	33.8	30.0	29.9	30.0
	Second	34.9	34.5	35.3	30.0	29.9	30.0
	Third	34.5	34.2	34.9	30.0	29.9	30.0
	Fourth	36.2	35.9	36.6	29.9	29.9	30.0
Top 50% per quarter for one year after the disaster	First	55.9	55.5	56.3	49.9	49.9	50.0
	Second	56.6	56.2	57.0	50.0	49.9	50.0
	Third	55.4	55.0	55.8	50.0	49.9	50.0
	Fourth	57.2	56.8	57.6	49.9	49.9	50.0

PM: Predictive Margin, CI: Confidence Interval, First quarter: July 2018 to September 2018, Second quarter: October 2018 to December 2018, Third quarter: January 2019 to March 2019, Fourth quarter: April 2019 to June 2019

Lastly, we show the results of GEEs that examined healthcare service utilization by disaster status and quarter (Table 4). The disaster status and interaction term between disaster status and quarter were all statistically significant (p < 0.001). Participants above 80 years of age had 1.28 times the service utilization rate compared to participants less than 20 years of age(outpatient care: 0.246 (p < 0.001), inpatient care: 2.238 (p < 0.001), dispensing pharmacy: 0.428 (p < 0.001). In addition, women used more outpatient care and dispensing pharmacies and used less inpatient care than men (outpatient care: 0.044 (p < 0.001), inpatient care: − 0.157 (p < 0.001), dispensing pharmacy: 0.025 (p < 0.001)). The PMs by quarter is listed in Table 5. For all health services, the risk of utilization was the highest immediately after the disaster. The difference in PMs between victims and non-victims was 7.0% (95% CI: 6.7–7.4), utilization of inpatient care was 1.3% (95% CI: 1.1–1.5), and dispensing pharmacy was 5.9% (95% CI: 5.5–6.4) in the first quarter after the disaster. In addition, this increase continued throughout the observation period.

**Table 4:** Generalized Estimating Equation model showing disaster status by health service utilization

		Outpatient care				Inpatient care				Medication			
		Coef.	Exp.	p value	SE	Coef.	Exp.	p value	SE	Coef.	Exp.	p value	SE
Disaster status	Non-victims	Reference				Reference				Reference			
	Victims	0.093	1.1	< 0.001	0.002	0.267	1.31	< 0.001	0.019	0.122	1.13	< 0.001	0.005
Age classification	0–19	Reference				Reference				Reference			
	20–39	-0.141	0.87	< 0.001	0.001	0.232	1.26	< 0.001	0.006	–0.252	0.78	< 0.001	0.001
	40–59	0.013	1.01	< 0.001	0.001	0.412	1.51	< 0.001	0.006	–0.009	0.99	< 0.001	0.001
	60–79	0.231	1.26	< 0.001	0.0005	1.416	4.12	< 0.001	0.005	0.329	1.39	< 0.001	0.001
	≥ 80	0.246	1.28	< 0.001	0.001	2.238	9.37	< 0.001	0.005	0.428	1.53	< 0.001	0.001
Sex, n (%)	Men	Reference				Reference				Reference			
	Women	0.044	1.04	< 0.001	0.0003	–0.157	0.85	< 0.001	0.003	0.025	1.03	< 0.001	0.001
Quarter for one year after the disaster	First	Reference				Reference				Reference			
	Second	0.023	1.02	< 0.001	0.0003	–0.009	0.99	< 0.001	0.002	0.063	1.07	< 0.001	0.0004
	Third	0.076	1.08	< 0.001	0.0003	–0.033	0.97	< 0.001	0.003	0.149	1.16	< 0.001	0.0004
	Fourth	0.031	1.03	< 0.001	0.0003	–0.025	0.98	< 0.001	0.003	0.07	1.07	< 0.001	0.0004
Interaction term between disaster status and a quarter	First	Reference				Reference				Reference			
	Second	–0.008	0.99	0.003	0.0028	–0.142	0.87	< 0.001	0.023	–0.026	0.97	< 0.001	0.004
	Third	–0.056	0.95	< 0.001	0.0029	–0.214	0.81	< 0.001	0.026	–0.084	0.92	< 0.001	0.005
	Fourth	–0.018	0.98	< 0.001	0.0029	–0.14	0.87	< 0.001	0.026	–0.043	0.96	< 0.001	0.005
Coef.: coefficient. Exp.: exponentiated parameter estimate, SE: standard error, First quarter: July 2018 to September 2018, Second quarter: October 2018 to December 2018, Third quarter: January 2019 to March 2019, Fourth quarter: April 2019 to June 2019													

**Table 5:** Predictive Margins on Quarterly Health Service Utilization (%)

	Quarter	Victims			Non-victims			
		PM (%)	95% CI		PM (%)		95% CI	
			Lower	Upper			Lower	Upper
Outpatient care	First	79.2	78.8	79.6		72.2	72.1	72.2
	Second	80.4	80	80.7		73.9	73.8	73.9
	Third	80.8	80.4	81.1		77.9	77.8	77.9
	Fourth	80.2	79.8	80.6		74.4	74.4	74.4
Inpatient care	First	5.5	5.3	5.7		4.2	4.2	4.2
	Second	4.7	4.5	4.9		4.1	4.1	4.2
	Third	4.3	4.1	4.5		4	4	4
	Fourth	4.6	4.4	4.8		4.1	4.1	4.1
Dispensing pharmacy	First	51.4	51	51.9		45.5	45.5	45.6
	Second	53.4	52.9	53.8		48.5	48.4	48.5
	Third	54.9	54.5	55.4		52.8	52.8	52.9
	Fourth	52.8	52.4	53.3		48.8	48.8	48.9

PM: Predictive Margin, CI: Confidence Interval, First quarter: July 2018 to September 2018, Second quarter: October 2018 to December 2018, Third quarter: January 2019 to March 2019, Fourth quarter: April 2019 to June 2019

## Discussion

This study revealed that the victims of the 2018 Japan Floods had higher medical costs than those who were not. In addition, the risk of becoming a high-cost patient was the highest at the end of the observation period. In comparison, healthcare service utilization increased immediately after the disaster. These elevated risks continued for one year after the disaster.

The total healthcare costs of victims became higher than those of non-victims in the period of one year after the 2018 Japan Floods, although there were no significant differences between them before the disaster. The attributable costs from the disaster were $355.3 (95% CI: 226.8–483.8). In the Great East Japan Earthquake (GEJE), healthcare costs increased. The changes in the total healthcare costs per capita were less than one-tenth of this estimation in an ecological study [34]. Because we directly identified people who sustained damage and were designated as victims, the results of this study can help estimate the increase in healthcare costs directly caused by future floods and torrential rains. The WHO has noted that the problem of healthcare costs is vital to building a healthcare system that is resilient against climate change, and there are few estimations of these costs [35]. We have already shown the change in costs of long-term care insurance for older victims of this disaster [17]. Putting these results together, we can provide a more comprehensive account of health-related cost changes for the victims. Governments and municipalities must estimate the increase in healthcare costs in response to the magnitude of such disasters and consider disaster preparedness.

The risk of becoming a high-cost patient was higher during the period over one year after the disaster, with the lowest risk in the first quarter and the highest risk in the fourth quarter. These results suggested that victims need medical care not only immediately after the disaster, but also continue to need medical care due to effects that impact long-term health. A previous study also showed that a natural disaster caused the deterioration of long-term health. The effects of the GEJE exacerbated the cognitive function and ADL of emigrated victims six years after the disaster [36]. In addition, the GEJE caused an increase in total long-term medical costs [37]. Disaster management and policies aimed at various social factors, such as community coping strategies, population relocation, compensation, and employment, influence long-term health status [38]. Considering these factors, long-term planning after a disaster is required for each region to address the exacerbated health status caused by torrential rains and floods.

Healthcare service utilization was more frequent among victims than non-victims during the observation period. In addition, the highest period of service utilization was in the first quarter of the 2018 Japan Floods. In contrast, because the GEJE resulted in extensive damage and destroyed healthcare facilities, service utilization diminished immediately after the disaster [34]. The recovery of utilization requires a few months. These differences in the initial situations for accessing healthcare services may be related to the type of disaster. The 2018 Japan Floods were mainly caused by local landslides or water exposure, and the damage was geographically limited [39]. Thus, healthcare facilities that were not damaged could accommodate the increased need for health services. For future disasters, healthcare facilities should respond to an expected increase in healthcare needs, considering the collaboration between each healthcare facility and sharing patient information.

Participants older than 80 years had higher service utilization, especially inpatient care, than other age groups. This trend is consistent with that of regular national censuses. Therefore, this result suggested that older people tended to need health services, especially inpatient care, than younger people as same as ordinal times even after disasters had occurred [40]. Meanwhile, women used inpatient care less than men. This finding is inconsistent with the census data [40]. The disaster could have created unusual and varied needs for inpatient care in the community. A nationwide survey of older individuals found fewer hospitalizations in women than in men [41]. The impact of a disaster occurs unevenly in the regional population. These factors were recognized as the causes of health care disparities [42, 43]. To reduce the future disparities, it would be required to strengthen the consistency and equity of care in publicly funded health plans [44].

This is the first study to examine changes in healthcare costs and service utilization caused by the 2018 Japan Floods using comprehensive data covering almost all medical facilities in the most affected prefectures. Accordingly, this study examined the overall effect of disasters on healthcare costs and service utilization. This database, which is managed by the national government, is highly precise. The similar policy of exempting victims from paying copayment for medical expenses has been adopted in subsequent floods and torrential rains. Therefore, the results of this study can be applied to other disasters in Japan as well.

Our study had several limitations. First, this study could not examine socio-demographic data, except for age and sex, specific diseases, severity, and outcomes of individual patients. A precise patient address was also not considered. Furthermore, the database does not classify medical institutions in terms of their size, department, content, and accessibility. For future studies, a database that includes these factors would make it possible to examine the disaster effect at a more individualized level for various factors than was possible in this study. Second, although we identified disaster victims based on receipt data, a certain number of people who were hurt or impacted by the disaster did not receive certification as a victim. Because these cases were included as non-victims, this study could have underestimated the actual effects of the disaster. Third, there were similar disasters in 2017 and 2019 during our study period, but the current cohort was not affected, and the affected regions were different from the Hiroshima, Okayama, and Ehime prefectures [12]. Therefore, the result of this study could not apply to situations where the torrential rains repeated within a certain period. Finally, the analysis in this study does not consider price elasticity. The price elasticity of medical care differs depending on the medical care. However, because we evaluated the effect on the healthcare system, including the increase in demand for medical care due to price elasticity, this study was able to show the comprehensive changes in medical costs and service utilization due to the 2018 Japan Floods.

## Conclusion

Victims of the 2018 Japan Floods had higher medical costs and used more healthcare services. In addition, the risk of higher medical costs was highest at the end of the observation period. In comparison, healthcare service utilization increased immediately after the disaster. It is necessary to estimate the increase in healthcare costs according to the disaster scale and plan for appropriate post-disaster healthcare service delivery. Governments and municipalities should promote disaster preparedness in vulnerable areas. Regional residents should also recognize and prepare for the risks of a disaster.

## Electronic supplementary material

Below is the link to the electronic supplementary material.

Supplementary Material 1

Supplementary Material 2

Supplementary Material 3

## Data Availability

The data that support the findings of this study are available from the Ministry of Health, Labour and Welfare, but restrictions apply to the availability of these data, which were used under license for the current study, and so are not publicly available. However, data are available from the authors upon reasonable request and with permission from the Ministry of Health, Labour and Welfare.

## List of abbreviations

GEEs: Generalized Estimating Equations
NDB: National Database of Health Insurance Claims
PMs: Predictive Margins
GEJE: Great East Japan Earthquake
