# Supplementary material for: Effects of 2018 Japan floods on healthcare costs and service utilization in Japan: a retrospective cohort study
Source: BMC Public Health. 2023 Feb 8;23:288. doi: 10.1186/s12889-023-15205-w (PMC9909853; doi:10.1186/s12889-023-15205-w)
Supplement: Supplementary file 1 — Supplementary Material 1 [file 12889_2023_15205_MOESM1_ESM.docx]

Supplementary Table 1: Cutoff Costs for Top 10%, 30% and 50% and Percentage of Consumed Total Medical Costs

|  | Quarter | Top 10% | Top 30% | Top 50% |
| --- | --- | --- | --- | --- |
| Cutoff costs (USD) | First | 1148.2 | 387.6 | 148.0 |
|  | Second | 1192.5 | 415.8 | 167.1 |
|  | Third | 1142.8 | 413.3 | 186.6 |
|  | Fourth | 1178.0 | 411 | 167.2 |
| Percentage of consumed total medical costs (%) | First | 73.0 | 90.8 | 97.7 |
|  | Second | 72.0 | 90.1 | 97.3 |
|  | Third | 71.1 | 88.8 | 96.3 |
|  | Fourth | 72.0 | 90 | 97.2 |
| First quarter: July 2018 to September 2018, Second quarter: October 2018 to December 2018, Third quarter: January 2019 to March 2019, Fourth quarter: April 2019 to June 2019 | | | | |
