# Supplementary material for: Effects of 2018 Japan floods on healthcare costs and service utilization in Japan: a retrospective cohort study
Source: BMC Public Health. 2023 Feb 8;23:288. doi: 10.1186/s12889-023-15205-w (PMC9909853; doi:10.1186/s12889-023-15205-w)
Supplement: Supplementary file 2 — Supplementary Material 2 [file 12889_2023_15205_MOESM2_ESM.docx]

Table 2: Results of Generalized Estimating Equations on Yearly Total Medical Costs

|  | | Coef | Exp | p value | SE |
| --- | --- | --- | --- | --- | --- |
| Disaster status | Non-victims | Reference | | | |
|  | Victims | -0.0094 | 0.99 | 0.628 | 0.019 |
| Age classification | 0-19 | Reference | | | |
|  | 20-39 | -0.073 | 0.93 | <0.001 | 0.005 |
|  | 40-59 | 0.645 | 1.91 | <0.001 | 0.004 |
|  | 60-79 | 1.56 | 4.76 | <0.001 | 0.004 |
|  | 80- | 2.13 | 8.42 | <0.001 | 0.006 |
| Sex | Men | Reference | | | |
|  | Women | -0.108 | 0.90 | <0.001 | 0.003 |
| Year | July 2017 – June 2018  (pre-disaster) | Reference | | | |
|  | July 2018 – June 2019  (post-disaster) | 0.092 | 1.10 | <0.001 | 0.001 |
| Interaction term between disaster status and a quarter | July 2017 – June 2018  (pre-disaster) | Reference | | | |
|  | July 2018 – June 2019  (post-disaster) | 0.120 | 1.13 | <0.001 | 0.016 |
| Coef: coefficient, Exp: exponentiated parameter estimate, SE: standard error | | | | | |
