# Supplementary material for: Effects of 2018 Japan floods on healthcare costs and service utilization in Japan: a retrospective cohort study
Source: BMC Public Health. 2023 Feb 8;23:288. doi: 10.1186/s12889-023-15205-w (PMC9909853; doi:10.1186/s12889-023-15205-w)
Supplement: Supplementary file 3 — Supplementary Material 3 [file 12889_2023_15205_MOESM3_ESM.docx]

Supplementary Table 3: Generalized Estimating Equation model showing disaster status by total medical costs

|  | | Top 10% | | | | Top 30% | | | | Top 50% | | | |
| --- | --- | --- | --- | --- | --- | --- | --- | --- | --- | --- | --- | --- | --- |
|  |  | Coef. | Exp. | p value | SE | Coef. | Exp. | p value | SE | Coef. | Exp. | p value | SE |
| Disaster status | Non-victims | Reference | | | | Reference | | | | Reference | | | |
|  | Victims | 0.081 | 1.08 | <0.001 | 0.012 | 0.108 | 1.11 | <0.001 | 0.006 | 0.113 | 1.12 | <0.001 | 0.004 |
| Age classification | 0-19 | Reference | | | | Reference | | | | Reference | | | |
|  | 20-39 | 0.12 | 1.13 | <0.001 | 0.006 | -0.177 | 0.84 | <0.001 | 0.003 | -0.23 | 0.79 | <0.001 | 0.002 |
|  | 40-59 | 0.94 | 2.56 | <0.001 | 0.005 | 0.589 | 1.80 | <0.001 | 0.001 | 0.286 | 1.33 | <0.001 | 0.001 |
|  | 60-79 | 2.04 | 7.69 | <0.001 | 0.005 | 1.426 | 4.16 | <0.001 | 0.002 | 0.825 | 2.28 | <0.001 | 0.001 |
|  | 80- | 2.75 | 15.64 | <0.001 | 0.005 | 1.816 | 6.15 | <0.001 | 0.002 | 0.991 | 2.69 | <0.001 | 0.001 |
| Sex, n (%) | Men | Reference | | | | Reference | | | | Reference | | | |
|  | Women | -0.15 | 0.86 | <0.001 | 0.002 | -0.031 | 0.97 | <0.001 | 0.001 | 0.14 | 1.15 | <0.001 | 0.001 |
| Quarter for one year after the disaster | First | Reference | | | | Reference | | | | Reference | | | |
|  | Second | -0.0003 | 1.00 | 0.81 | 0.001 | -0.0003 | 1.00 | 0.53 | 0.001 | 0.00004 | 1.00 | 0.91 | 0.0004 |
|  | Third | -0.0004 | 1.00 | 0.90 | 0.001 | -0.0003 | 1.00 | 0.64 | 0.001 | 0.0001 | 1.00 | 0.81 | 0.0004 |
|  | Fourth | -0.001 | 1.00 | 0.30 | 0.001 | -0.0006 | 1.00 | 0.26 | 0.001 | -0.0002 | 1.00 | 0.65 | 0.0004 |
| Interaction term between disaster status and a quarter | First | Reference | | | | Reference | | | | Reference | | | |
|  | Second | 0.033 | 1.03 | 0.008 | 0.012 | 0.044 | 1.04 | <0.001 | 0.006 | 0.011 | 1.01 | 0.003 | 0.004 |
|  | Third | 0.018 | 1.02 | 0.18 | 0.013 | 0.034 | 1.03 | <0.001 | 0.006 | -0.009 | 0.99 | 0.021 | 0.004 |
|  | Fourth | 0.13 | 1.14 | 0.11 | 0.16 | 0.083 | 1.09 | <0.001 | 0.006 | 0.022 | 1.02 | <0.001 | 0.004 |
| Coef: coefficient, Exp: exponentiated parameter estimate, SE: standard error, First quarter: July 2018 to September 2018, Second quarter: October 2018 to December 2018, Third quarter: January 2019 to March 2019, Fourth quarter: April 2019 to June 2019 | | | | | | | | | | | | | |
